# TIHI Toolkit: A Peak Finder and Analyzer for Spectroscopic Data

**DOI:** 10.1021/acsomega.4c06830

**Published:** 2024-12-06

**Authors:** Kyunghoon Han, Ariadni Boziki, Alexandre Tkatchenko, Joshua T. Berryman

**Affiliations:** Department of Physics and Materials Science, University of Luxembourg, L-1511 Luxembourg City, Luxembourg

## Abstract

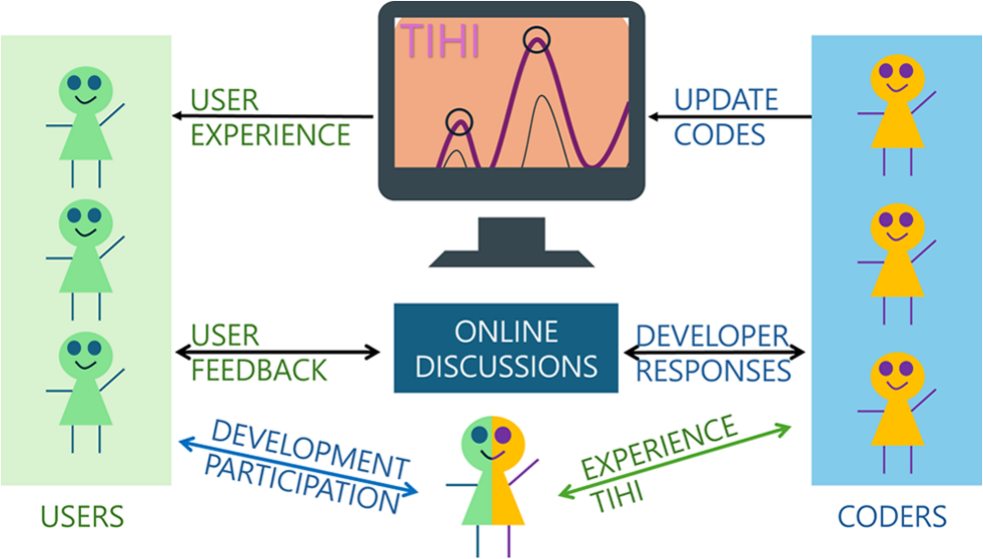

Complex signal vectors, particularly spectra, are integral to many scientific domains. Interpreting these signals often involves decomposing them into contributions from independent components and subtraction or deconvolution of the channel and instrument noise. Despite the fundamental nature of this task, researchers frequently rely on costly commercial tools. To make such tools accessible to all, we present *Tihi*, interactive, open-source multiplatform software for interpolation, denoising, baseline correction, peak detection, and signal decomposition. *Tihi* provides a user-friendly graphical interface (GUI) that facilitates the analysis of spectroscopic data and more. It allows researchers to contribute to and freely distribute these tools, ensuring broad accessibility and fostering collaborative improvements. We present examples demonstrating the efficiency of the program using the spectra of different systems acquired by different spectroscopic techniques, including Raman (aspirin), IR (solid ammonia), XRD (anatase), and UV–vis (petal tip from the *Puya alpestris* flower). These examples showcase a variety of spectra that differ significantly, from signals with narrow profiles to signals with very broad profiles. This demonstrates the versatility of *Tihi* for peak identification in a wide range of spectroscopic techniques.

## 1 Introduction

Spectroscopic techniques are essential tools for revealing the structure, composition, and behavior of matter. The analysis of characteristic peaks in a spectrum provides critical insights into the chemical properties of a system. For example, in mass spectroscopy, these peaks offer precise information about the chemical composition, while in vibrational spectroscopy, they help identify functional groups present in a molecule. Similarly, the examination of absorption peaks in various spectroscopic methods provides valuable data on the molecular structure and overall composition of materials. The accuracy of peak detection has a significant impact on the results. However, experimental signals often contain random noise, alternating baselines, varying peak shapes, and overlapping peaks, making it crucial to use a method that can reliably detect peaks in the spectra.

Several peak detection methods have been developed, including the direct peak-locating algorithm,^1,2^ first and second derivative techniques,^3−5^ curve fitting,^6−8^ the Fourier transform method,^9^ and the wavelet transform method.^10−16^ Among these, the wavelet transform-based algorithm has gained considerable attention in recent years due to its accuracy, performance and multiscale nature. However, all of these peak detection methods encounter difficulties when applied to spectral regions with closely spaced peak pairs, entire peak clusters, or features exhibiting a high dynamic range. Such complex spectral features are common in molecular systems like large proteins and periodic materials.^17^ In recent years, advances in machine learning, particularly the rise of deep neural networks, have opened new avenues for developing models that can more accurately identify spectral peaks.^17−20^ However, to the best of our knowledge, each model remains tailored to a specific spectroscopic technique, and no universal model exists that can handle the peaks of spectra across all types of spectroscopy.

Spectroscopists frequently rely on commercial software options for analyzing and interpreting spectral data. Popular choices include Spectragryph, Mnova, OriginLab, LabView, Opus as well as routines in platforms like Matlab, Octave, Excel and OpenOffice among others.^21−27^ These tools offer a comprehensive suite of functionalities for data analysis. Typically provided features include baseline correction, smoothing, normalization, peak fitting, and spectral deconvolution, the latter of which helps separate overlapping peaks to reveal underlying molecular or elemental contributions.^28−35^ Additionally, updating these tools to meet the evolving needs of the scientific community can be challenging and does not allow user-driven changes.^10,36,37^ While the premium versions of these software solutions provide extensive capabilities, their high cost can limit accessibility, making it challenging for many researchers and institutions within the spectroscopy community to utilize them effectively.

As alternatives to commercial software, there are several free online tools available that can analyze spectral data effectively. These tools often deliver performance comparable to paid versions, as demonstrated by ChemoSpec.^38^ Examples of such tools include Vernier Spectral Analysis, ChemInformer, and SpecVizPro, among others.^39−41^ However, these free online tools typically depend on server-side computations with no clear way to modify the algorithm on the user side, rendering them ineffective when the user is obliged to embed a new tool into the provided software. This limitation is also problematic for outdoor experiments or for analysis embedded directly in data-gathering devices (remote sensing). Additionally, updating these tools to meet the evolving needs of the scientific community can be challenging and do not allow user-driven changes.

Both commercial and open-source spectroscopic analysis software offer a range of features, but they are often specialized for specific types of data. For instance, certain tools are designed exclusively for NMR spectral data (e.g.,TopSpin),^42^ while others focus on IR and Raman spectra (e.g.,Opus, DiscovIR10 Software, GIRAS),^25,43,44^ and some to mass spectroscopy (e.g.,MaxQuant, MALDIquant, TIPick, NITPICK).^2,45−47^ Such specializations are natural for their purposes, as the characteristic peaks and intensities in various spectra are tied to different molecular properties. Focusing on a single spectroscopic technique, combined with additional features like band assignment and peak decomposition, these tools can achieve more precise peak detection. However, these specialized tools rely on mathematical methods that can be adapted to any type of signal. Thus, the specialization should not obscure the fact that the underlying techniques are fundamentally general-purpose, making cross-application and interoperability both feasible and, in many cases, desirable. Ideally, these specialized features should function as add-ons to a versatile, general-purpose platform that can handle a broader range of spectroscopic data.

Here, we introduce, *Tihi*†, a user-friendly graphical user interface (GUI) tool for peak detection and signal decomposition in spectroscopic data analysis. This tool features a minimalist design for enhanced user clarity and ease of use, incorporates a modular architecture for easy updates and feature modifications, and is accessible to the community, allowing for adaptations to meet the diverse needs of scientists.

Our publication consists of four main sections. Section 2 details detrending algorithms, peak-identification methods, and the decomposition of signals into a sum of distributions. In Section 3, we delve into the graphical interface, highlighting its features and functionalities. Notably, the software comes with a stepwise tutorial for utilizing denoising and refinement methods effectively. While our software prioritizes accessibility and community-driven updates, advanced alternative methods briefly mentioned in Section 2.1 are yet to be implemented, where simpler methods like linear, airPLS and arPLS methods are implemented.^48^ In Section 4, we showcase the applicability of *Tihi* on different spectroscopic signals. We conclude by discussing the advantages offered by our application.

## 2 Methodology

The peak identification workflow is designed to systematically process spectroscopic data, which often contains complex signals. Accurate peak identification and quantification are crucial for understanding a sample’s composition. The workflow typically begins with an optional step of removing any unwanted trends in the signal, known as detrending or baseline correction. This ensures that the analysis focuses on true spectral features, eliminating artifacts introduced by the instrument or sample preparation.

After detrending, the next step is to identify potential peaks in the spectrum using a technique called window propagation. This method scans the signal with a moving window to pinpoint regions where peaks are likely present, helping to distinguish closely spaced features more effectively. Once these candidate peaks are detected, the workflow moves on to modeling the signal component around each peak using mathematical distributions, such as Gaussian, Lorentzian or Voigt distributions. The goal is to fit these distributions such that their combined signal closely reproduces the original (detrended) spectrum, allowing for more accurate quantification of overlapping peaks. Finally, the processed spectrum and corresponding peak data are saved for further analysis, allowing for a detailed interpretation of the chemical properties of the system under study.

### 2.1 Signal Detrending Algorithms

Signal detrending (here including baseline correction), aims to correct bias in the captured signal at coarse scales, by removing an error signal which is constant or varies slowly on the scale of the structure in the data. Accurate decomposition of finer structure in spectral data typically begins with alignment of the baseline to the *x*-axis. Intuitive recognition of the true baseline of the signal is often easy, but a rigorous algorithmic approach is needed in order to have sensible, or at least consistent, results in difficult cases.

Mathematically, we define a “baseline” as a function , where
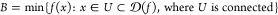
1Here,  and  represent the domain and range of the function or distribution *f*. It is important to note that *B* is not unique based on this definition. What we ascertain from this definition is that *f*(*x*) – *B*(*x*) yields a function that measures the deviation or alteration from the initial value of interest.

Since baselines are not unique, various types of baseline correction algorithms exist, including, linear, Shirley backgrounds,^49,50^ penalized least-squares (PLS)/Tikhonov method,^51^ polynomial fittings, derivative methods, CROWELL,^52^ LIMPIC,^53^ and corner-cutting,^54^ to name a few. In this section, and in the program itself, we focus on three simple algorithms: linear, adaptive iteratively reweighted PLS (airPLS),^55^ and asymmetrically reweighted PLS (arPLS).^56^

A selection of algorithms are implemented in *Tihi*: the linear baseline algorithm is the simplest approach. It comprises drawing a straight line from the initial coordinates to the signal’s end-point. This method is both fast and less susceptible to distorting the signal’s shape. However, researchers might seek a nonlinear baseline or may aim to reshape the signal to remove noise from the profile. This is where PLS algorithms prove beneficial. These algorithms are efficient, easy to debug, and flexible enough to accommodate nonlinear baselines and refine signal shapes during denoising processes.

The “PLS” algorithms airPLS, arPLS, establish the baseline as a vector denoted as **z** following Baek et al.^56^ This is achieved through minimizing the regularized least-squares function, or the cost function, defined as
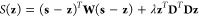
2Here, **D** represents the difference matrix:
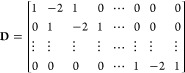
3**s** represents the signal under analysis, while the matrix **W** and the scalar λ serve as parameters for fine-tuning. The fitness of the data and the smoothness of the baseline are represented in the first and second terms of eq 2, respectively. For airPLS, the selection among different least-squares methods is determined by the choice of the parameters in the matrix **W** = [*w*_*i*_]_*i*_.
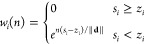
4where *n* is the iteration step, *s*_*i*_ is the *i*-th component of the signal, *z*_*i*_ is the *i*th component of the baseline, and **d** is the negative elements of **s** – **z**. The rationale behind the weight selections in this method is 2-fold: first, to preserve whatever is already above the baseline, and second, to iteratively update weights exponentially to extend into the region beyond the baseline when *s*_*i*_ falls below it.

The arPLS algorithm is expressed below, defining the parameters **W** as follows
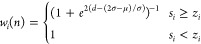
5Here, σ represents the standard deviation, and μ signifies the mean of the negative values of **s** – **z**. Unlike airPLS, this method endeavors to guide the logistic function toward convergence at 1, when *s*_*i*_ ≥ *z*_*i*_. This adjustment ensures that the majority of baseline values lie beneath the signal, resulting in a primarily positive detrended signal. As a consequence, this method yields results closer to scientists’ expectations from spectroscopic data.

**Figure 1:**
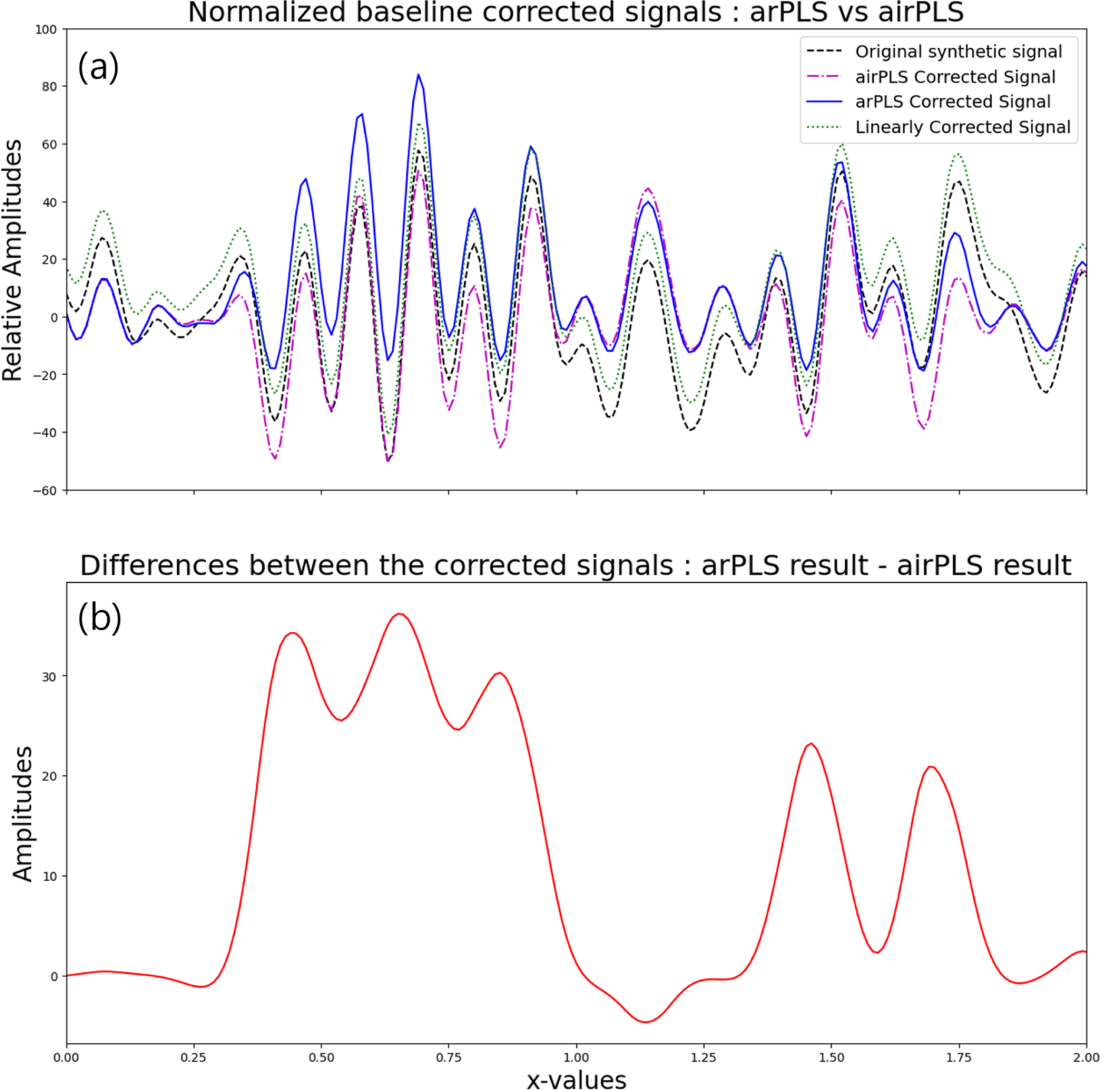
(a) Comparison between the original signal (generated using multiple sine functions), the linearly detrended signal, and signals detrended using airPLS and arPLS methods. (b) Difference between the arPLS and airPLS results, showing the differences that can arise from the two baseline correction methods. Note that the positions of the peaks are not altered despite the changes in amplitudes. The original signal is generated to emphasize an extreme case.

Figure 1 compares the linear baseline correction method with the two PLS-based algorithms discussed in this section. The linear method is computationally efficient and preserves the shape of the signal but cannot eliminate nonlinear trends. In contrast, as shown in Figure 1a, the arPLS algorithm adjusts the baseline to keep the signal as positive as possible, while airPLS does not impose this constraint. Consequently, arPLS is more suitable for broader signals with multiple overlapping distributions, whereas airPLS is better suited for signals that are inherently non-negative.

### 2.2 Peak Detection Method

Peak detection by window propagation entails a two-step procedure:

1. Define the local interval, called the **window**, within the signal.
2. Identify the local maximum of the signal within the defined window. This maximum is determined among potential candidates where the negative of the second derivative of the signal reaches a local maximum.

The window is propagated (slid) along the *x*-axis. If a candidate peak, defined as the local maximum (second derivative minimum), is found, a list of potential candidates is constructed. These candidates are then filtered using a threshold that defines the ideal size of the peak of interest. A signal may consist of multiple overlapping components, forming shoulders rather than distinct peaks: use of second derivatives is necessary in this circumstance.

**Figure 2:**
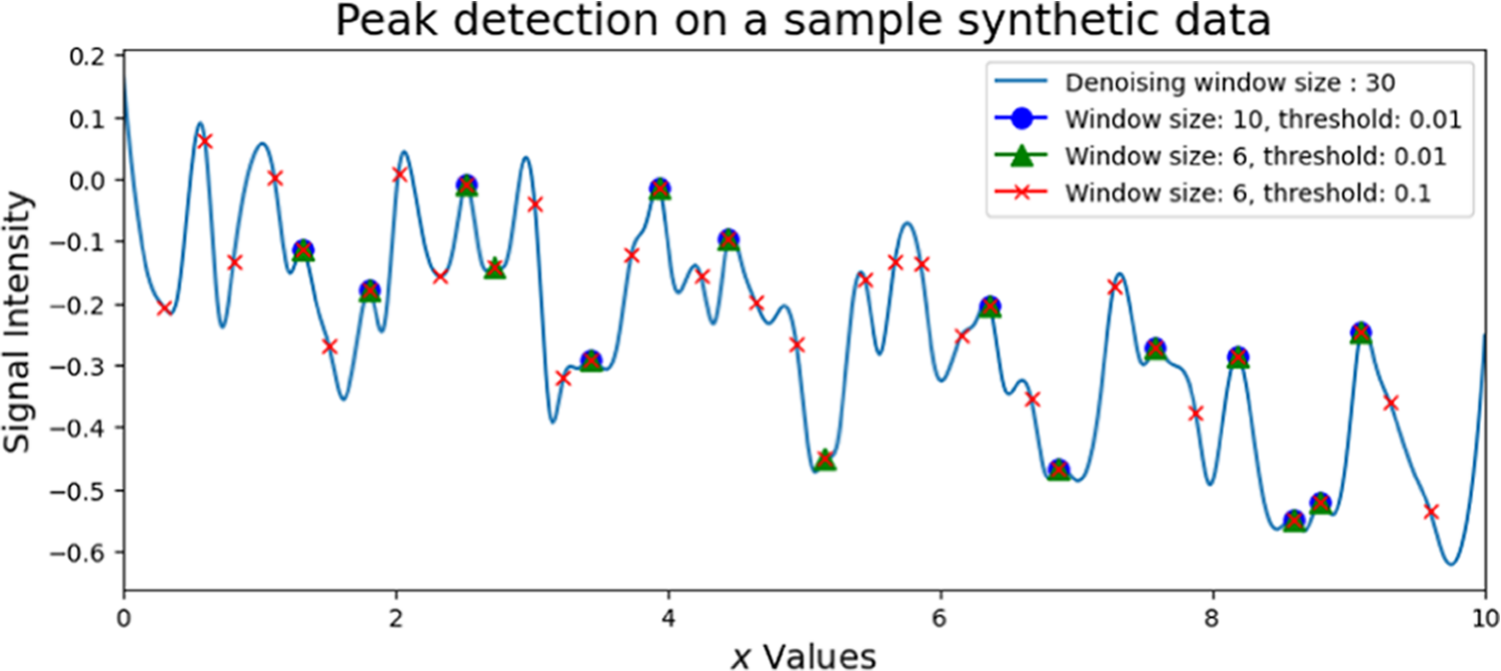
A test signal with varying window parameters. A smaller window size and a higher threshold result in a greater number of potential peaks. However, a smaller window or lower threshold also increases the likelihood of including spurious peaks generated by noise.

Results from window propagation are sensitive to the choice of window size and the degree of overlap between neighboring windows. A smaller window size generates more candidate peaks from the signal compared to a larger window size. This is due to the increased likelihood of the amplitude corresponding to the median of the window reaching its maximum in a smaller window. Users are required to set a threshold based on the difference between the maximum and minimum amplitudes within the specified interval. This threshold helps filter out peaks with insignificant heights, thereby improving the accuracy of the analysis. Figure 2 illustrates the outcomes obtained using various sliding window sizes and thresholds, highlighting the benefits of interactively selecting the optimal window size. Smaller window sizes and lower thresholds tend to identify more peak candidates, which increases the risk of false positives. For instance, the first peak in Figure 2, marked with a red cross (window size = 5, threshold = 0.1), demonstrates how minor local fluctuations can be mistakenly classified as peaks. Conversely, if the window sizes are too large or the thresholds are set too small, one may not find enough peaks to accurately capture the underlying physics or chemistry of the model.

### 2.3 Decomposition into Multiple Distributions

The primary objective of the tasks detailed in the preceding two subsections is to generate a series of distributions that can be added to form the corrected signal. The measured input signal is thus accounted for as a sum of physically meaningful independent signals (plus the baseline correction). Many chemical or biological signals can be economically represented as sums of well-defined distributions, often with Gaussian, Lorentzian/Cauchy, or Voigt functional forms.

Assume a denoised and detrended signal, *ŝ*, with peaks identified. The first working definition of a peak is as a local maximum with height above the nearest stationary point exceeding a certain threshold, taken as a parameter. Peaks showing not as maxima but as shoulders in the input signal can be detected iteratively following this scheme, once the dominant peaks are recognized. The task our program needs to accomplish to obtain the decomposition is to find the parameters, **p**, that satisfy the following equality.
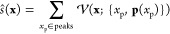
6where  can be Gaussian, Lorentzian, or Voigt distribution. Note that the parameters depend on the peaks, *x*_p_, because the distributions corresponding to the different peaks have distinct shapes and sizes.

Similar to baseline correction, the parameters defining the distributions are determined by optimizing a specific cost function. The simplest function for optimization involves measuring the discrepancy between the targeted sum and the original signal. It can be represented as
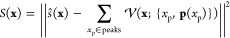
7The objective is to minimize *S*(**x**), ideally achieving a value of zero. Although reaching zero is highly improbable, the aim is to get as close as possible, especially if the signal *ŝ* maintains positive definiteness and continuity within a bounded set. In spectroscopic applications, this set usually represents an interval where the signal is defined. Determining the parameters involves identifying the minima of *S* by computing the partial derivatives of *S* with respect to the parameters ∂*S*/∂**p**. This well-known optimization approach is commonly referred as the least-squares optimization algorithm.

The least-squares algorithm offers a user-friendly approach to optimization, ensuring efficiency and simplicity. Its roots date back to 1722 at the latest, with Roger Cotes,^57^ and its utility was demonstrated by Legendre and Laplace in their astronomical predictions.^58^ Gauss later formalized it, establishing it as an optimal and well-understood method we know today.^59^ Despite its advantages, the community retains the flexibility to opt for alternative optimization algorithms.

**Figure 3:**
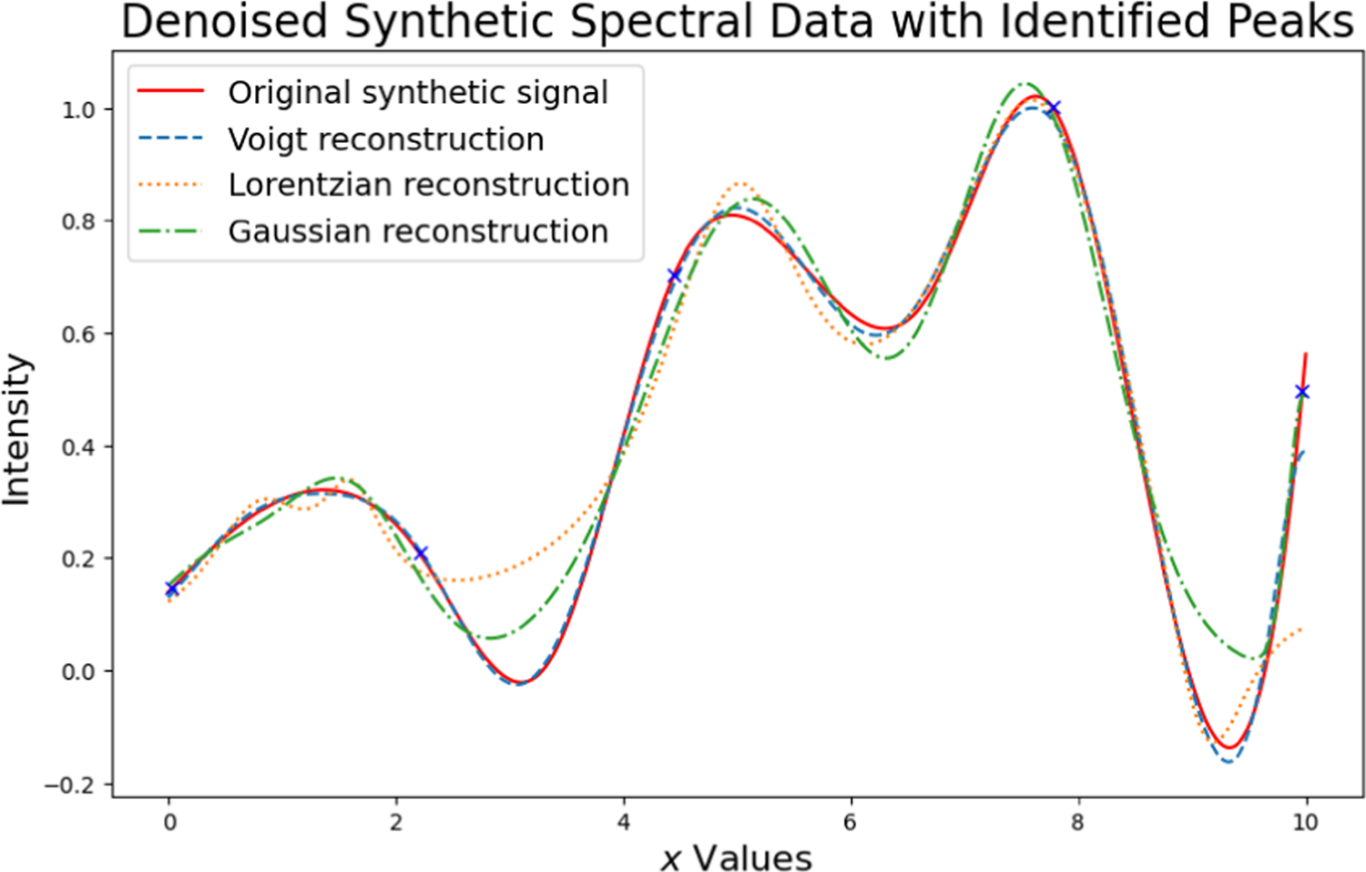
Demonstrating the fitting of spectral data with diverse distributions involves using the least-squares method to optimize the distribution parameters. This process contributes to accurately reconstructing the original signal.

Figure 3 presents a synthetic spectral signal alongside its identified peaks and reconstructions using Voigt, Lorentzian, and Gaussian distributions. The original synthetic signal is compared with these reconstructions to evaluate their accuracy. Among the three, the Voigt reconstruction provides the closest fit to the original signal across most of the spectrum. In contrast, the Lorentzian reconstruction exhibits some deviations, particularly in the valleys between peaks, where it tends to overestimate the intensity. The Gaussian reconstruction, while generally aligning with the trend of the original signal, underestimates peak heights and overestimates valley depths in certain regions. This comparison illustrates the importance of choosing an appropriate distribution for accurate signal reconstruction in spectral analysis.

## 3 Features of the Code

**Figure 4:**
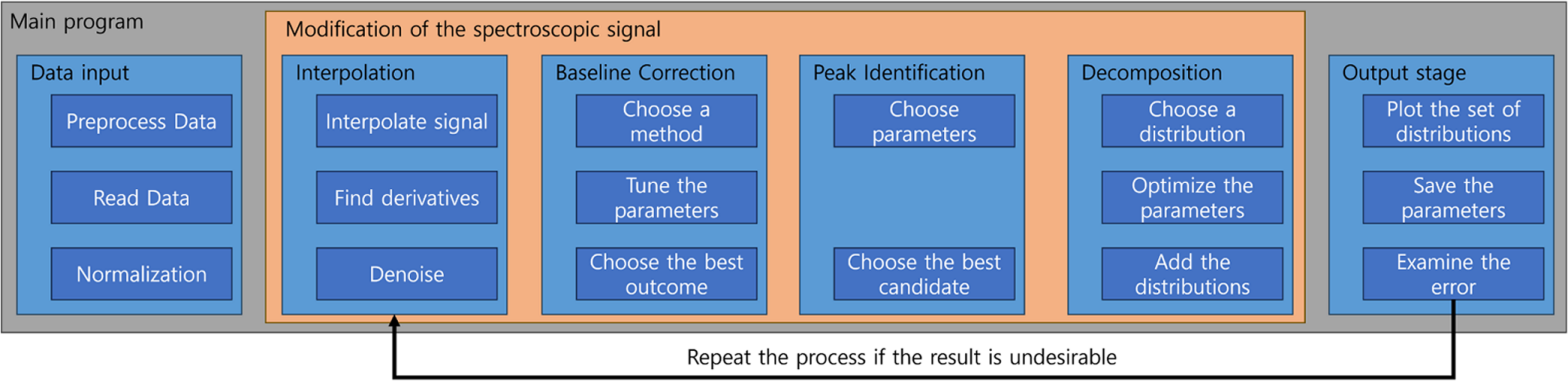
Illustrating the workflow for peak identification and distribution optimization. The features outlined in the orange box are implemented in wizard-style interface, as each step must follow the order and heavily relies on the previous results.

While the methods outlined in Section 2 may appear distinct at first glance, they converge toward a common objective; identifying peaks and distributions to construct a physically accurate signal. Figure 4 visually illustrates the strategic alignment of these methods, demonstrating the systematic extraction of precise peak information from spectroscopic data sets. *Tihi*’s open-source nature and deliberate modular design enhance its adaptability for future community-driven enhancements and facilitate streamlined debugging processes. The program is easy to use, maintain, and update. It can run locally with Python 3 installed. Additionally, its modularity allows for easy integration with C, Rust, Java, Javascript, or PHP code, making it suitable for embedding into hardware or web pages.

### 3.1 Main Section: Basic UI for Visualizing the Input Signal

**Figure 5:**
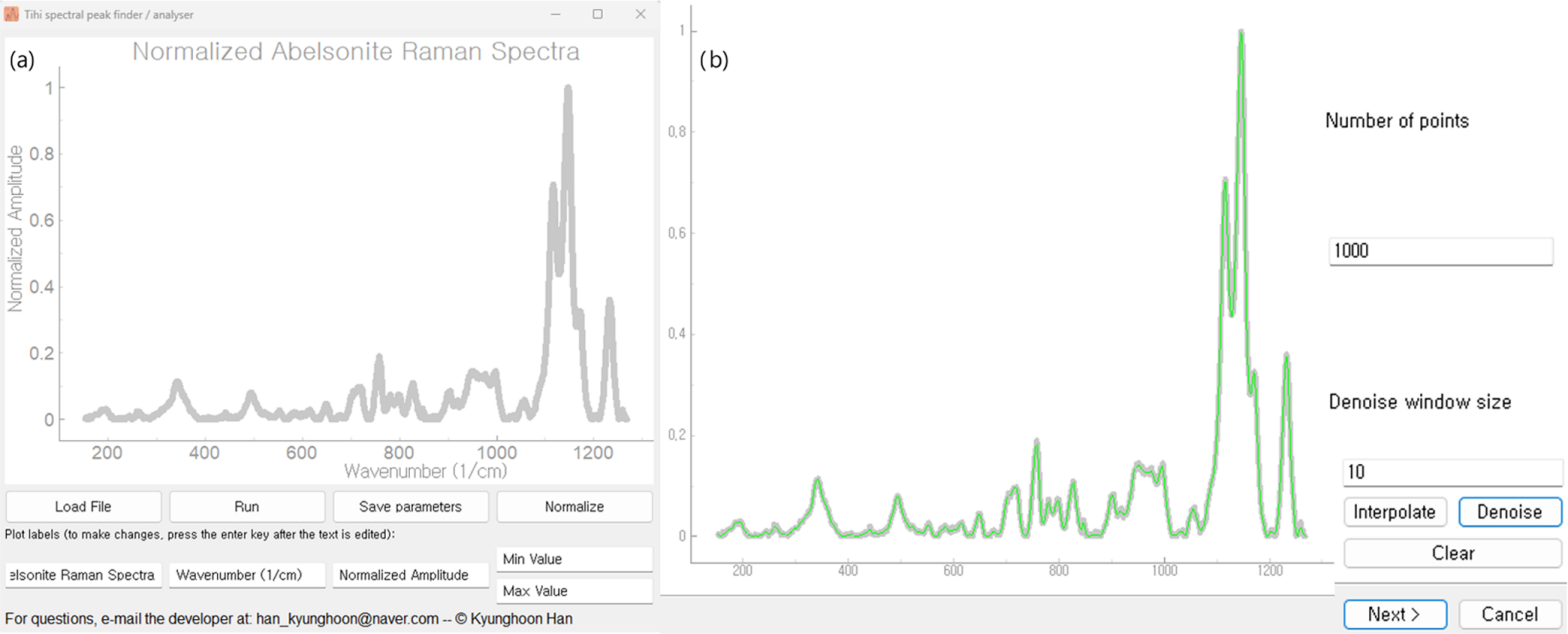
(a) The main window of *Tihi* offers a clean and straightforward interface, featuring a minimal selection of buttons: load, run algorithm, save parameters, normalize plot and set minimum and maximum values of the *x*-axis. Additionally, users can customize plot labels, conveniently. Data can be saved as a text file or exported as an image using the built-in functionality of the PyQtGraph library,^60^ accessible via a right-click on the plot. The showcased sample plot is sourced from the RRUFF database.^61^ (b) The wizard window appears when the user presses Run. It presents the denoised input signal, represented by the green curve. The buttons and labels of this image are magnified for visibility.

The program’s core functionality centers on two pivotal tasks: (1) managing the input and output of signal data and (2) displaying visual representations of preprocessed and modified results. The main window is designed to effectively carry out these tasks while maintaining user intuitiveness by minimizing the number of buttons and interactive menus. Figure 5 illustrates how the UI is structured to guide users with limited selections, preventing confusion or difficulty in navigation.

To demonstrate how to use *Tihi*, we use the Raman spectral signal of an abelsonite sample from the RRUFF database as a test case,^61^ shown in Figure 5. This signal is chosen for its complexity arising from abelsonite’s crystal structure that consists of nearly planar, covalently bonded porphyrin molecules held together by weak intermolecular van der Waals forces,^62^ showcasing *Tihi*’s full functionality even with the current backbone GUI program. Users can press Load File to import two-column data in .txt format. This step will immediately plot the spectral data, treating the first column as the *x*-axis.

### 3.2 Wizards: The Main Analysis

**Figure 6:**
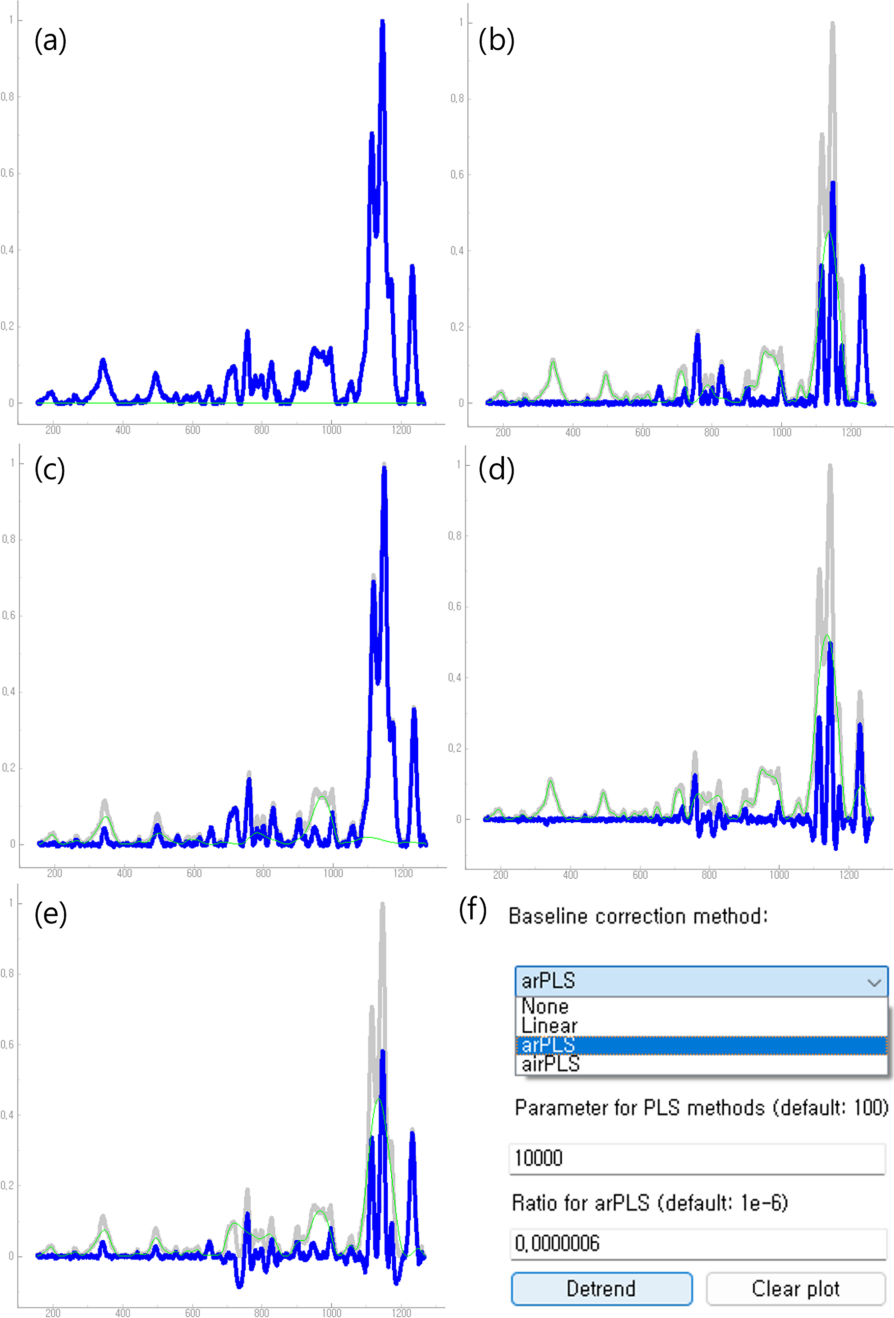
Application of different baseline correction algorithms with various smoothness parameters on the abelsonite Raman spectrum. Green lines indicate the baselines, blue lines show the corrected signal, and gray lines represent the original signal. (a) Linear baseline correction result, (b, c) arPLS baseline correction results with λ = 10^3^ and 10^4^, respectively, (d, e) airPLS baseline correction results with λ = 10^3^ and 10^4^, respectively, (f) menu interface for toggling options in the baseline correction window.

When users press Run in the main window, they are directed to a pop-up window containing the interpolation and denoising section of the program. Similar to other wizard windows in the program, this section is straightforward, with users only required to choose parameters and observe how their signal is denoised—refer to Figure 5 for the illustrated explanation. It is important to note that users are not immediately directed to the one-step peak detection and signal decomposition page. They are, instead, guided through a series of steps in the correct order to achieve the desired decomposition. This sequential approach is supported by the wizard UI, which encourages users to progress through the algorithm step-by-step. Even if users prefer to run the algorithm in reverse order, such as from decomposition to peak identification, this could pose mathematical challenges or be time-consuming. The wizard UI is designed to prevent users from encountering such difficulties and ensures a smoother experience. Upon clicking Next, users are directed to a window where they can select and apply the baseline correction algorithm. In this section, they make only three selections: the type of baseline correction algorithm, the smoothness parameter, λ, from eq 2 for PLS methods, and the ratio for the airPLS method. The results obtained from this step are shown in Figure 6. The optimal magnitude of the smoothness parameter depends on the signal’s nature and noise level. The GUI’s default value of 200 is not ideal for signals with many features, such as the abelsonite Raman spectrum. As shown in Figure 6c, a value of λ ∼ 10^4^ using the arPLS method works best, preserving features and successfully detrending the signal. This conclusion is based on a small set of trials. For less complex signals, the default value of 200 may suffice.

**Figure 7:**
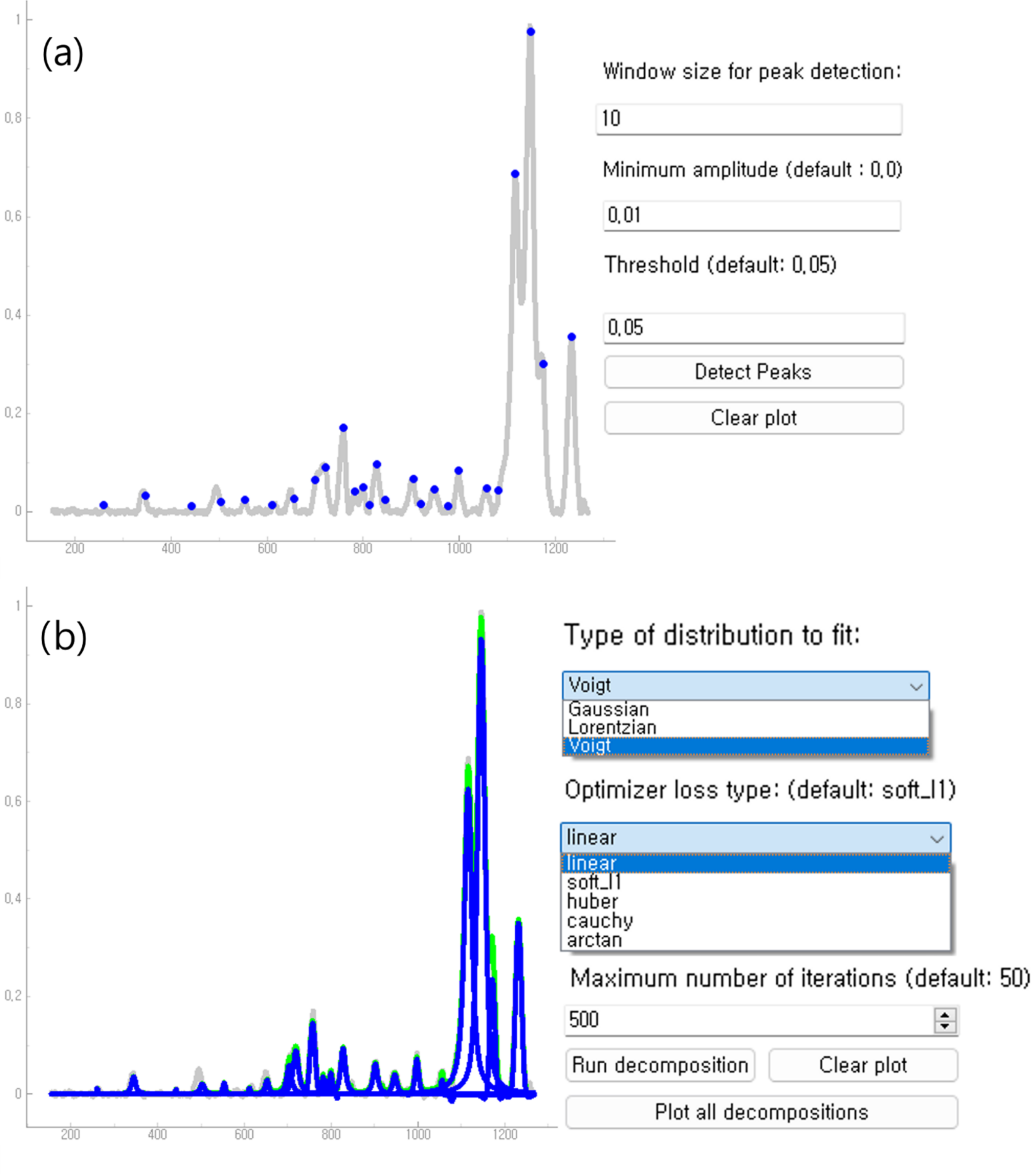
(a) Peak detection window following the baseline correction step. The UI is minimalistic, with only two parameters to choose: window size and the threshold. (b) Signal decomposition into multiple Lorentzian distributions. Users can choose from three distributions: Gaussian, Lorentzian, and Voigt, and select the optimization loss for the least-squares method from linear, soft l1, Huber loss, and arctan. The blue curves show the individual decompositions where the light green curve shows their sum.

After completing the signal detrending step, users proceed to a page to fine-tune their peak detection scheme, (see Figure 7a). On this page, three options are available: window size, threshold and minimum amplitude. The first two parameters are as they discussed in Section 2, while the minimum amplitude eliminates small peaks irrelevant to the extracted information. In cases where the signal is obtained from an experimental device, small noise-induced bumps may appear, making this parameter essential for noisy signals.

**Figure 8:**
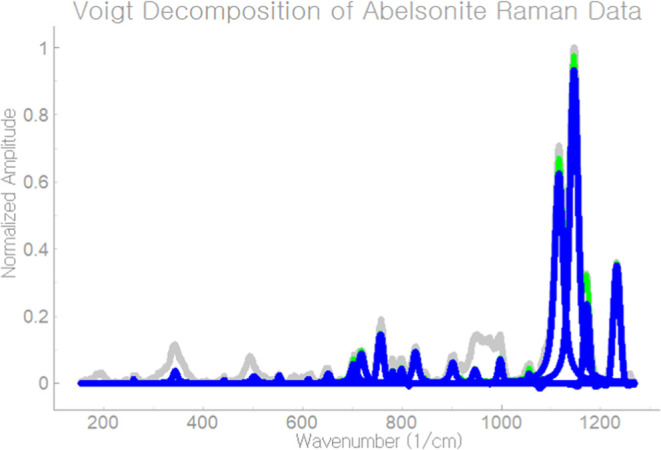
Program displays the final result on the main window of *Tihi*.

Once the peaks are identified, users can proceed to the next step; the signal decomposition page. This page consists of a plotting window and options similar to those in the previous pages. There are three options to choose from the type of distribution, optimizer loss, and maximum number of iterations (see Figure 7b). Pressing Run decomposition initiates the decomposition process. Once the process is completed, the final fitted result will be displayed on the graphing window. It is important to note that this process will run the decomposition using the parameter set obtained from the previous run. This allows users to examine intermediate fitted results in units of the Maximum number of iterations each time they click this button. Once the decomposition is terminated, one can examine the individual distributions on the graphing window by clicking Plot all decompositions and obtain a graph as in Figure 8.

## 4 Results and Discussion

To verify the effectiveness of our code, *Tihi*, in peak detection of spectral signals, we demonstrated its capabilities using a variety of spectra from different spectroscopic techniques. While it is impractical to showcase every type of spectroscopy, we selected four diverse examples: the experimental Raman spectrum of aspirin, our simulated IR spectrum of solid ammonia, the XRD spectrum of anatase and the UV–vis spectrum of the petal tip from the *Puya alpestris* flower. These spectra were chosen because they vary significantly in signal characteristics, from having narrow profiles to very broad profiles. This diversity highlights the efficiency of our program in identifying peaks in spectra with different properties. Our code is versatile and can be used for peak identification in any spectroscopic spectrum.

### 4.1 Raman Spectroscopy

**Figure 9:**
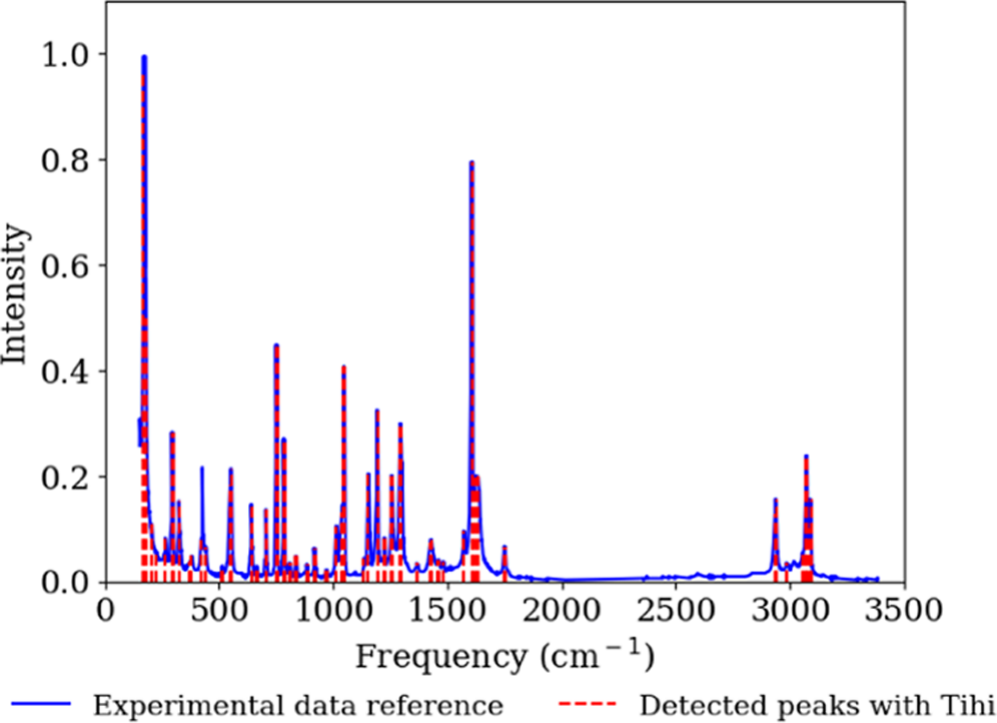
Experimental Raman spectrum of aspirin (blue line), retrieved from the SpectraBase database,^63^ compared with *Tihi*’s detected peaks and in turn vibrational frequencies (red line). The optimized *Tihi* settings were as follows: the original signal was interpolated with 10,000 data points, and the denoising window was set to 8. The arPLS algorithm with λ = 200 and a ratio of 1 × 10^–6^ was used for baseline correction. The window size for peak detection was 10, with a minimum amplitude of 0.01, and a threshold of 5 × 10^–6^. Lorentzian distribution was selected and the optimization loss for the least-squares method was set to soft l1, with a maximum of 100 iterations.

We retrieved the experimental Raman spectrum of aspirin from the SpectraBase database.^63^Figure 9 shows the Raman spectrum of aspirin along with the detected peaks by *Tihi*, indicated by vertical lines, using optimized parameters. These parameters include the baseline correction method and window size for peak detection, as described in Section 3 among others. At first glance, our code with optimized parameters appears capable in accurately predicting the peak values and corresponding vibrational frequencies of aspirin. However, for a more accurate assessment, we compare *Tihi*’s detected peaks and in turn frequencies with those reported in the literature for aspirin.

Our goal is to showcase the efficiency and accuracy of our program. Comparing frequencies based on various previous studies, which likely used different experimental settings, can be misleading and does not allow a direct comparison of our code’s effectiveness. Therefore, we focus solely on the results related to this particular spectrum, even though not all bands are assigned to specific vibrations. In the experimental reference spectrum, aromatic rings are observed at 1030 cm^–1^, and the C–O–H vibration is observed at 1200 cm^–1^. The C–CH_3_ vibration appears at 1300 cm^–1^, while the carbonyl group (C=O) shows a stretching vibration at 1600 cm^–1^. Vibrations associated with C–H are observed at 2950 cm^–1^ and those with O–H are at 3050 cm^–1^.^64^ As shown in Table 1, our detected vibrational frequencies compare well with the experimental ones. The differences observed in some frequencies can be justified by the unknown accuracy of the algorithm used to detect the experimental frequencies. If the methods were not highly accurate, such deviations are expected.

**Table 1:** Performance Benchmark of *Tihi* for Raman Spectrum of Aspirin^a^

frequency reported^63,64^ (cm^–1^)	frequency detected with *Tihi* (cm^–1^)
1030	1038
1200	1196
1300	1299
1600	1610
2950	2942
3050	3054

a This table lists the experimental frequencies (peak positions) assigned to specific vibrations,^63,64^ alongside the corresponding frequencies detected by *Tihi*.

### 4.2 IR Spectroscopy

**Figure 10:**
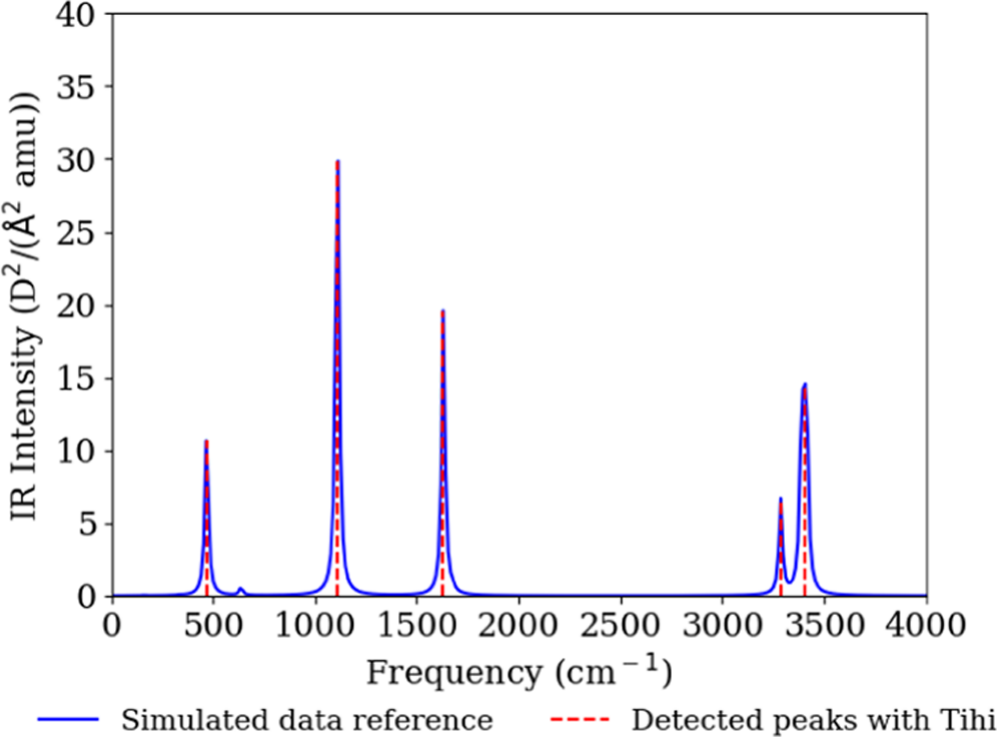
Simulated IR spectrum of solid ammonia (blue line), compared with *Tihi*’s detected peaks and in turn vibrational frequencies (red line). The optimized *Tihi* settings were as follows: the original signal was interpolated with 10,000 data points, and the denoising window was set to 10. The arPLS algorithm with λ = 200 and a ratio of 1 × 10^–6^ was used for baseline correction. The window size for peak detection was 18, with a minimum amplitude of 0.022, and a threshold of 0.009. Lorentzian distribution was selected and the optimization loss for the least-squares method was set to soft l1, with a maximum of 100 iterations.

We simulated IR spectral data of solid ammonia, in Figure 10. Due to the broadening process, the peaks may deviate from the actual vibrational frequencies–explaining the deviation shown in Table 2 despite the excellent agreement.

**Table 2:** Performance Benchmark of *Tihi* for the Simulated IR Spectrum of Solid Ammonia^a^

frequency in sim. data (cm^–1^)	frequency detected with *Tihi* (cm^–1^)
468	469
1112	1111
1631	1631
3287	3286
3405	3412

a This table lists the simulated frequencies assigned to specific vibrations, alongside the corresponding frequencies detected by *Tihi*. Abbreviation: sim.: simulated reference data.

As shown in Figure 10, the simulated IR spectrum of ammonia is presented along with the peaks detected by *Tihi* indicated by vertical lines. Specifically, the peaks at 468, 1112, 1631, 3287 and 3405 cm^–1^ correspond to lattice mode ν_L_, symmetric bending ν_2_, antisymmetric bending ν_4_, symmetric stretching ν_1_, and a combination of symmetric stretching ν_1_ and lattice mode ν_L_, respectively. With optimized settings parameters, the program accurately predicts the peak positions. Table 2 further illustrates this agreement by listing the vibrational frequencies of the main bands, corresponding to the individual lines before spectral broadening, alongside the peak positions detected by *Tihi*. Except for the high frequency band, all other frequencies show excellent agreement between the actual simulated vibrational frequencies and those estimated by our code. The deviation in the high frequency band can be attributed to the broadening method applied to the individual lines of frequencies and IR intensities, as mentioned earlier. Since *Tihi* analyzes the broadened spectrum, such a shift in frequency may result from the broadening process and should not be considered a flaw in the program.

### 4.3 X-ray Diffraction (XRD)

For the XRD analysis, we selected the XRD spectrum of anatase (TiO_2_) due to its extensive industrial applications, including the production of plastics, artificial fibers, electronic materials, rubber and solar cells.^65,66^ This spectrum is characterized by sharp peaks, differing from the other spectra we have examined. Consequently, identifying the peaks using *Tihi* for the entire spectrum at once is not optimal. To address this, we divided the spectrum into different *x*-axis windows: [20,50], [50,60], [60,68], [68,72] and [72,85], and estimated the peaks within each window.

**Figure 11:**
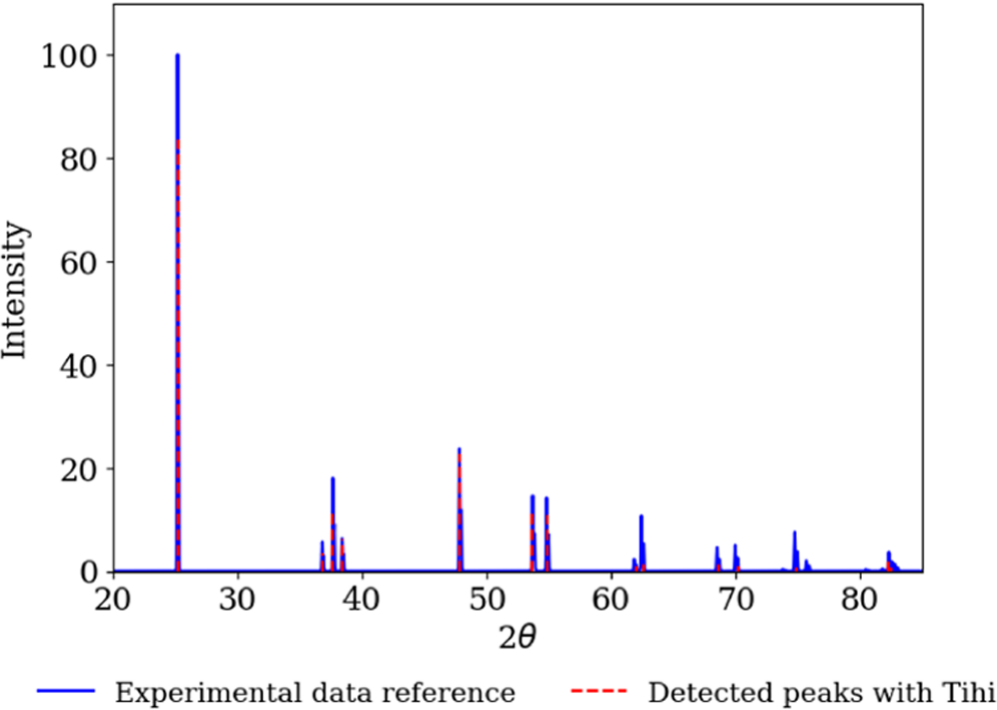
XRD spectrum of anatase (blue line),^61,67^ compared with the peaks detected by *Tihi* (red line). To identify the peaks with *Tihi*, the spectrum was divided into different *x*-axis windows. The optimized *Tihi* settings for each window were as follows: The original signal was interpolated with 1000 data points for all *x*-axis windows. No denoising and detrending methods were applied. The window size for peak detection was 18. The minimum amplitude settings were 0 for windows with ranges [20,50], [50,60] and [68,72], 0.01 for the window with range [60,68], and 0.1 for the window with range [72,85]. The threshold was set to 0.05. Gaussian distribution was selected and the optimization loss for the least-squares method was set to linear, with a maximum of 50 iterations for all windows except the window with range [68,72], which was set to 100 iterations.

In Figure 11, we present the experimental spectrum of anatase,^61,67^ along with the peaks detected with our program. We observe a very good agreement between the estimated and experimental peaks. To further verify the accuracy of our program in identifying the peaks of the XRD spectrum, we calculated the Miller indices (*h*, *k* and *l*), for the peaks detected by *Tihi* and compared them with the reported values (see Table 3). We utilized Bragg’s law for this calculation:
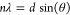
8where *n* is the diffraction order (usually *n* = 1 for XRD analysis), θ is the angle of incidence, *d* is the grating distance and λ is the wavelength of the incident X-rays. Given that anatase has a tetragonal crystal structure with lattice constants *a*, *b* and *c*, where *a* = *b* ≠ *c*, the interplanar spacing d_*hkl*_ for a tetragonal crystal system is given by
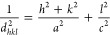
9Combining eqs 8 and 9, we can determine the Miller indices corresponding to the peaks in the XRD spectrum by solving for the possible combinations of *h*, *k* and *l* using the equation for *d*_*hkl*_ in a tetragonal system:
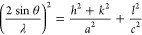
10

**Table 3:** Performance Benchmark of *Tihi* for the XRD Spectrum of Anatase^61,67^^,^^a^

exp. 2θ^61,67^	ref Miller indices (*h k l*)^61,67^	2θ	Miller indices (*h k l*)
25.23	1 0 1	25.24	1 0 1
36.86	1 0 3	36.80	1 0 3
37.72	0 0 4	37.66	0 0 4
38.46	1 1 2	38.43	1 1 2
47.89	2 0 0	47.86	2 0 0
53.77	1 0 5	53.70	1 0 5
54.89	2 1 1	54.87	2 1 1
61.92	2 1 3	62.02	2 1 3
62.51	2 0 4	62.57	2 0 4
68.59	1 1 6	68.59	1 1 6
70.05	2 2 0	70.24	2 2 0
74.83	2 1 5	74.85	2 1 5
75.78	3 0 1	75.54	3 0 1
82.41	2 2 4	82.28	2 2 4
82.87	3 1 2	82.52	3 1 2

a This table compares the experimental angles of incidence θ and their corresponding Miller indices (*h k l*) with the angles of incidence and Miller indices detected by *Tihi*. Abbreviations: Exp.: experimental angle of incidence, ref: reference data.

We used a wavelength of λ = 1.54 Å, typical for Cu Kα radiation, to calculate the Miller indices. As shown in Table 3, the peak positions detected by our program exhibit excellent agreement with the experimental peaks. The calculated Miller indices also match well with the reported values, demonstrating that our program can accurately identify peaks in signals with sharp features, such as the XRD spectrum of anatase.

To evaluate *Tihi*’s peak detection performance, we analyzed the XRD spectrum of anatase using two other widely used peak detection tools (see Table 1 in the Supporting Information). *Tihi* successfully identified all the peaks in the spectrum. Similarly, Spectragryph^21^ also performed well, although it missed one peak. In contrast, OriginPro,^23^ detected 13 out of 15 peaks, but its deviations from the reference data were more significant compared to both *Tihi* and Spectragryph.

### 4.4 Ultraviolet–Visible (UV–vis) Spectroscopy

To evaluate the effectiveness of *Tihi* in UV–vis spectroscopy, we analyzed the UV–vis spectrum of the petal tip from the *Puya alpestris* flower,^68^ which grows at lower elevations on the western side of the Andes in Central Chile. The distinctive blue-green pigment in *Puya alpestris* is identified as a nonacylated anthocyanin, delphinidin 3,7,3′-tri-*O*-glucoside. This anthocyanin is known to decolorize rapidly in an aqueous solution at pH 5.3–5.5. Therefore, various spectroscopic methods, including UV–vis spectroscopy, are crucial for investigating the chemical factors responsible for the green-blue coloration in Puya species.

**Figure 12:**
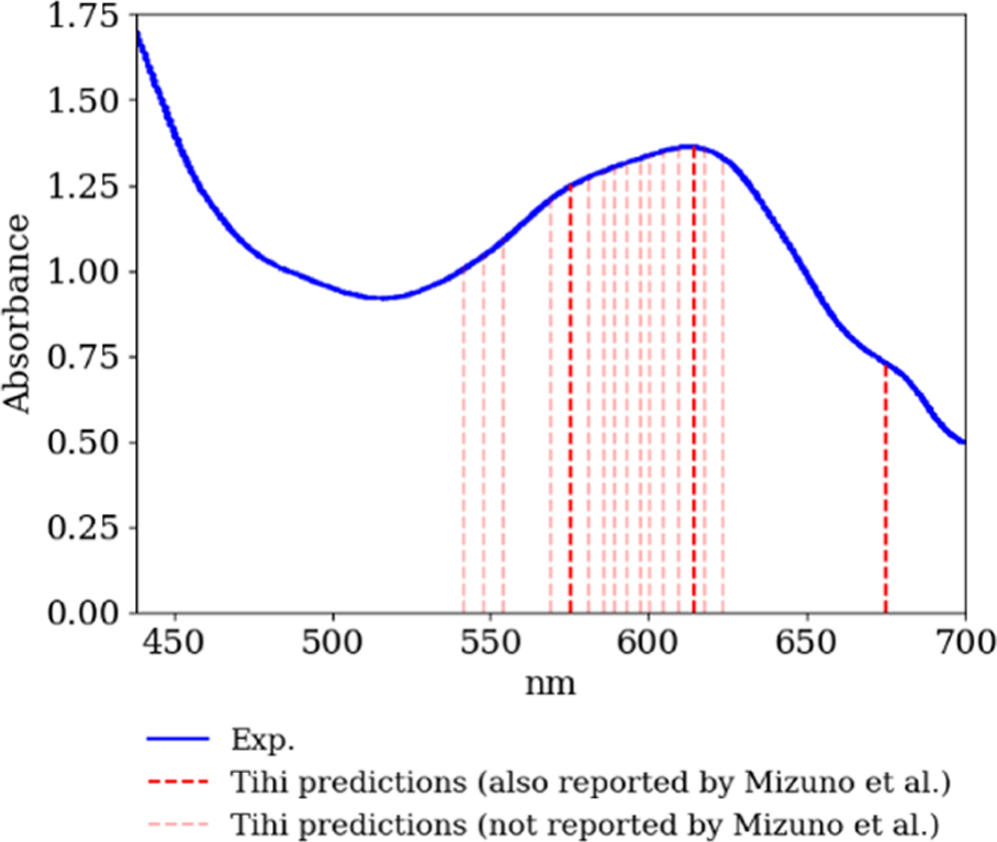
UV–vis absorption spectrum of the petal tip of *Puya alpestris* from,^68^ compared with the peaks detected by *Tihi* (red). The dark red lines indicate peaks also reported by Mizuno *et. al*,^68^ while lighter red indicates additional peaks identified by the program. The optimized *Tihi* settings were as follows: the original signal was interpolated with 1000 data points, and denoising was applied three times with sizes of 100, 30 and 10, consecutively. The detrending method was not used. The window size for peak detection was set to 10, with a threshold of 0.01. A Lorentzian distribution was selected and the optimization loss for the least-squares method was set to linear, with a maximum of 100 iterations.

Figure 12 presents the experimental UV–vis absorption spectrum of the petal tip, highlighting three absorption maxima at 575, 614, and 679 nm. These maxima are shown alongside the peaks identified by our program. Mizuno et al.^68^ attributed the absorption maximum at 679 nm to chlorophyll and the maxima at 575 and 614 nm to the anthocyanin. To demonstrate our program’s accuracy, the peaks it identified that correspond to the experimentally observed ones are marked in dark red, while the others are indicated in light red.

As a first glance, the UV–vis spectrum appears smooth, suggesting that peak identification might be straightforward. However, this is not the case; it is actually a challenging system to analyze due to spectral broadening. The difficulty arises from the varying full width at half-maximum (fwhm) used for the distributions that sum to produce the spectrum. There can be scenarios where a few distributions with larger fwhm result in fewer peaks, or more distributions with narrower fwhm result in more peaks.

We hypothesize that the reason our program predicted more peaks than those previously reported for the UV–vis spectrum is due to the use of narrower fwhm compared to those used by Mizuno et al.^68^ This discrepancy should not be considered a flaw in our implementation, as it depends solely on the settings used in the previous peak identification. Despite this condition, as shown in Table 4, our program accurately identified the three peaks reported by Mizuno et al.^68^ These findings illustrate *Tihi*’s capability to accurately identify peaks in spectra with significant broadening and even propose additional peaks, offering insights into the pigment composition of *Puya alpestris* flower.

**Table 4:** Performance Benchmark of *Tihi* for UV–vis Absorption Spectrum of the Petal Tip from the *Puya alpestris* Flower^68^^,^^a^

frequency reported^68^ (nm)	frequency detected with *Tihi* (nm)
575	576
614	615
679	675

a This table lists the experimental frequencies, alongside the frequencies detected by *Tihi*.

Additionally, similar to the analysis of the XRD spectrum of anatase, we assessed *Tihi*’s performance in predicting the spectral peaks of the UV–vis spectrum of the petal tip of *Puya alpestris* flower, compared to other tools, (see Table S2 of Supporting Information). Our findings indicate that *Tihi* accurately predicts multiple peaks, whereas other tools either detect only a single peak or exhibit higher deviations from the reference data.

## 5 Conclusions

Here we introduce *Tihi*, an open-source user-friendly GUI tool designed for peak detection and signal decomposition in spectroscopic data analysis. *Tihi* offers a comprehensive backbone workflow for signal processing that includes baseline correction, peak detection and signal decomposition, ensuring high flexibility and precision. Its minimalist design enhances clarity and ease of use, while the modular architecture allows for easy adaptation and future enhancements. Users can efficiently manage their data, visualize results, and fine-tune parameters for optimal signal reconstruction. The program’s practicality is further enhanced by the ability to save and export data. Additionally, with the entire code available online, users can modify the software independently and run their applications locally without relying on a web server. Importantly, the open-source nature of the program invites researchers worldwide to enhance the tool, ensuring more scientists have access to a local peak decomposition GUI tool on their desks.

Our program is equipped with the least-squares optimization algorithm, coupled with various baseline correction methods (linear, airPLS, arPLS), so that it can effectively refine an input signal. The wizard UI approach ensures that users follow a structured, step-by-step process, minimizing errors and enhancing the accuracy of peak detection and signal decomposition. To showcase the efficiency of our program, we used both publicly available experimental spectroscopic data and our own simulated spectroscopic data from different techniques, including Raman (aspirin), IR (solid ammonia), XRD (anatase) and UV–vis (petal tip from *Puya alpestris* flower). These spectra were chosen because they represent a wide range of signal characteristics, from spectra with narrow profiles to spectra with very broad profiles. These examples demonstrate the program’s success in peak identification, highlighting its versatility in analyzing spectroscopic data of any kind.

Future work may involve integrating additional signal detrending and optimization algorithms to broaden the scope of the application. Given the rapid advancements in machine learning techniques, we will explore their potential for peak identification, particularly in automating parameter selection and addressing complex signal patterns. While traditional algorithms have been prioritized for their interpretability and computational efficiency, machine learning approaches could help overcome challenges such as adaptive baseline correction and noise reduction. Enhancing parallelization capabilities could further decrease computation time for large data sets, which is critical for real-time applications.

Additionally, we aim to introduce add-on packages such as a peak assignment helper, 3D visualizer, and vibration visualizer. By continuously adapting and improving our code based on user feedback, we will address issues like limited configurability and complex error handling. Future versions of *Tihi* are expected to feature specialized wizard windows tailored to specific needs, simplifying the user experience for both novice and advanced users.

### 5.1 Computational Details

The IR spectrum of solid ammonia was calculated using the all-electron numeric-atom-centered orbital code FHI-aims (Fritz Haber Institute ab initio molecular simulations).^69−72^ This computation employed the PBE functional,^73^ enhanced by the nonlocal many-body dispersion (MBD-NL) method.^74^ The tight species default settings in FHI-aims were used for all numerical atom-centered basis functions and integration grids, incorporating scalar relativistic effects via the zero-order regular approximation (ZORA).

Convergence criteria were set to 10^–6^ eV for total energy, 10^–7^ electrons/Å^3^ for charge density, 10^–5^ eV/Å for the sum of eigenvalues, and 10^–4^ eV for forces. Geometry and cell relaxation was considered converged when the maximum residual force component per atom was below 10^–5^ eV/Å, with the maximum acceptable energy increase per relaxation step also set to 10^–5^ eV. The Brillouin zone was sampled with a 4 × 4 × 4 Monkhorst–Pack *k*-points grid.^75^ For the graphical representation of the vibrational spectra, Lorentzian broadening was applied with a full width at half-maximum (fwhm) set to 10.0.

## Data Availability

*Tihi* application is freely distributed via its GitHub repository: https://github.com/kyunghoon-han/tihi. *Tihi* is also distributed via PyPI: https://pypi.org/project/Tihi-spectral-fitter/.^76^ The data produced by *Tihi* is given in a Zenodo repository: https://zenodo.org/records/12570689.

## Supporting Information Available

Comparison of *Tihi*’s performance with other widely used tools for peak identification (PDF)

## Footnotes

† Tihi means “luck” in Greek, “peak” in Ma̅ori, and “silences” in Bulgarian and is an expression for acting cute in Korean.
