# Supplementary material for: TIHI Toolkit: A Peak Finder and Analyzer for Spectroscopic Data
Source: ACS Omega. 2024 Dec 6;9(50):49397–410. doi: 10.1021/acsomega.4c06830 (PMC11656381; doi:10.1021/acsomega.4c06830)
Supplement: Supplementary file 1 — ao4c06830_si_001.pdf [file ao4c06830_si_001.pdf]

TIHI Toolkit: A Peak Finder and Analyser for Spectroscopic Data

Kyunghoon Han[a],* Ariadni Boziki[a], Alexandre Tkatchenko[a], and Joshua T. Berryman[a]

[a]*Department of Physics and Materials Science, University of Luxembourg, L-1511, Luxembourg City, Luxembourg.*

E-mail: kyunghoon.han@proton.me

Table S1: Performance comparison between *Tihi*, Spectragryph[1] and OriginPro[2] for the XRD spectrum of anatase.[3,4] This table lists the experimental angles of incidence (θ), the angles detected by each tool, their absolute differences, and the percentage error relative to the experimental data. Missing data is indicated by asterisks (*). Abbreviations: Exp. for experimental, SG for Spectragryph.

Exp. Data	Tihi Results	Tihi Errors	SG Results	SG Errors	Origin Results	Origin Errors
25.23	25.24	0.01 (0.04%)	25.22	0.01 (0.04%)	24.08	1.15 (4.56%)
36.86	36.80	0.06 (0.16%)	36.83	0.03 (0.08%)	36.22	0.64 (1.74%)
37.72	37.66	0.06 (0.16%)	37.69	0.03 (0.08%)	37.96	0.24 (0.64%)
38.46	38.43	0.03 (0.08%)	38.43	0.04 (0.10%)	*	*
47.89	47.86	0.03 (0.06%)	47.85	0.04 (0.08%)	48.37	0.52 (1.09%)
53.77	53.70	0.07 (0.13%)	53.72	0.05 (0.09%)	53.57	0.20 (0.37%)
54.89	54.87	0.02 (0.04%)	54.85	0.05 (0.09%)	55.31	0.42 (0.77%)
61.92	62.02	0.10 (0.16%)	61.87	0.05 (0.08%)	60.51	1.41 (2.28%)
62.51	62.57	0.06 (0.10%)	62.46	0.06 (0.10%)	62.24	0.27 (0.43%)
68.59	68.59	0.00 (0.00%)	68.53	0.06 (0.09%)	*	*
70.05	70.24	0.19 (0.27%)	69.99	0.06 (0.09%)	70.91	0.86 (1.23%)
74.83	74.85	0.02 (0.03%)	74.76	0.07 (0.09%)	74.39	0.44 (0.59%)
75.78	75.54	0.24 (0.32%)	75.70	0.08 (0.11%)	76.12	0.34 (0.45%)
82.41	82.28	0.13 (0.16%)	82.33	0.08 (0.10%)	81.32	1.09 (1.32%)
82.87	82.52	0.35 (0.42%)	*	*	83.06	0.19 (0.23%)

Average Errors	0.09 (0.14%)	0.05 (0.09%)	0.60 (1.21%)

Table S2: Performance comparison between *Tihi*, Spectragryph[1] and OriginPro[2] for the UV-Vis absorption spectrum of the petal tip of *Puya alpestris*[5] flower. This table presents the peaks identified by Mizuno *et al.*,[5] alongside the peaks detected by *Tihi*, Spectragryph and OriginPro, including their absolute differences and percentage errors relative to the experimental data. Missing data are indicated by asterisks (*). Abbreviations used: Exp. for experimental, SG for Spectragryph.

Exp. Data	Tihi Results	Tihi Errors	SG Results	SG Errors	Origin Results	Origin Errors
575	576	1 (0.17%)	*	*	535	40 (6.96%)
614	615	1 (0.16%)	610	4 (0.65%)	612	2 (0.33%)
679	675	4 (0.59%)	*	*	688	9 (1.33%)

Average Errors	2 (0.31%)	4 (0.65%)	17 (2.87%)

References

(1) Spectragryph: optical spectroscopy software. `https://www.effemm2.de/spectragryph/`, version v1.2.16.1, 2022; Accessed on June 3rd, 2024.

(2) Origin(Pro): OriginLab Corporation. `https://www.originlab.com/`, version 2022; Accessed on June 3rd, 2024.

(3) Lafuente, B.; T., D. R.; Yang, H.; Stone, N. In *The power of databases: the RRUFF project.*; Armbruster, T., Danisi, R. M., Eds.; W. De Gruyter, 2015; pp 1–30.

(4) Horn, M. S. C. F.; Schwebdtfeger, C. F.; Meagher, E. P. Refinement of the structure of anatase at several temperatures. *Z. Kristallogr. Cryst. Mater.* **1972**, *136*, 273–281.

(5) Mizuno, T.; Sugahara, K.; Tsutsumi, C.; Iino, M.; Koi, S.; Noda, N.; Iwashina, T. Identification of anthocyanin and other flavonoids from the green–blue petals of Puya alpestris (Bromeliaceae) and a clarification of their coloration mechanism. *Phytochemistry* **2021**, *181*, 112581.
